# Psychosocial risk and protective factors in school victimization: an explanatory model in adolescents

**DOI:** 10.1186/s41155-025-00376-9

**Published:** 2026-02-05

**Authors:** Esperanza Vargas Jiménez, Sara Paola Pérez-Ramos, Remberto Castro Castañeda

**Affiliations:** Centro Universitario de la Costa, Universidad de Guadalajara, Av. Universidad , 48280 Puerto Vallarta, México

**Keywords:** School victimization, Life satisfaction, Psychological distress, Resilience, Social support, Adolescence, Secondary school, Structural Equation Modeling

## Abstract

**Background:**

School victimization during adolescence is a multidimensional phenomenon that affects psychological well-being and social development. Understanding its underlying factors is essential for designing effective preventive strategies.

**Objective:**

This study aims to analyze school victimization in adolescents from a psychosocial perspective, proposing an explanatory model that integrates individual variables (psychological distress, life satisfaction, and resilience) and social variables (community social support).

**Method:**

The total of participants was 1,687 adolescents (46% male, 54% female) aged 12–17 years (M = 13.65, SD = 1.14), from 13 schools in Puerto Vallarta, Mexico.

**Results:**

Psychological distress is positively associated with school victimization, while life satisfaction is negatively associated. Furthermore, community social support and resilience were found to indirectly influence school victimization through life satisfaction and, negatively, through psychological distress. Multigroup analysis revealed differences in victimization dynamics by gender, although the model is invariant.

**Conclusion:**

The findings highlight the importance of adopting a multidimensional perspective in understanding and addressing school victimization. Promoting resilience and strengthening community social support could reduce the risk of victimization and improve students' psychological well-being. These results have practical implications for the development of comprehensive prevention and intervention strategies in school settings.

## Introduction

The importance of a study focused on school victimization and its psychosocial variables lies in its high prevalence and the serious consequences it can have on well-being. According to the United Nations Children's Fund (UNICEF, 2018), half of adolescents worldwide have experienced some form of violence in the school setting. According to the Network for Children's Rights in Mexico (REDIM for its abbreviation in Spanish 2023), 28% of adolescents in the country reported having been victims of bullying in the previous 12 months, representing 3.3 million students.

According to UNESCO (2024), school bullying is not a simple conflict but a harmful social process where an individual or group with more power repeatedly engages in unwanted behavior. This power dynamic is influenced by social norms and the school environment. Bullying causes physical, emotional, and social harm not only to the victim but also to the entire educational community.

In essence, it is a manifestation of school violence that goes beyond individuals, rooted in power dynamics and societal norms. Bullying has been identified as a public health problem of increasing relevance (UNICEF, 2019), affecting adolescents regardless of school grade, socioeconomic background, or community setting. Although it commonly occurs among peers of the same age group, it implies an imbalance of power based on physical, emotional, or social differences (García Piña & Posadas Pedraza, 2018).

Roles in bullying are usually grouped into aggressors, victims, and observers (Mendoza González et al., 2022). This study focuses on victims, who are subjected to aggression in different forms, such as verbal, physical, and relational (Cedeño Sandoya, 2020). Forms of victimization include insults and humiliation (verbal victimization), beatings and threats (physical victimization), and social exclusion (relational victimization), generating psychological and social imbalances.

Bronfenbrenner's Ecological Systems Theory (1979) provides an analytical framework that is consistent with UNESCO's interdimensional view of school bullying. This theory posits that human development is intrinsically linked to the dynamic interaction between an individual and their environment.

From this viewpoint, school victimization cannot be reduced to an individual phenomenon. Its study must encompass the complex interactions among multiple environmental levels, including students, their families, educational institutions, public policies, and the sociocultural contexts that perpetuate or mitigate violence.

In this regard, various studies expose psychological distress as a direct consequence of school victimization, characterizing it as a state of emotional discomfort that includes different symptoms, such as sleep disturbances, psychosomatic conditions, and depressive symptoms (Balluerka, et al., 2022; Gini et al., 2024; Park, 2025), conditions that reinforce vulnerability to future episodes of bullying (Moore et al., 2017; Vega-Cauich & Euan-Catzin, 2020). These findings allow us to hypothesize that a cycle exists where distress predisposes to victimization and victimization aggravates distress. The present research offers an original approach by focusing on the Mexican population, a group still little explored in this field.

Another relevant variable in the study of school victimization is social support, understood as the set of emotional, informational, or material resources that a person perceives from their environment. It has been shown to be an important protective factor, since it not only attenuates the negative effects of victimization by promoting psychological well-being (Bartolomé Gutiérrez & Díaz Herráiz, 2020; Gini et al., 2024; Larrucea-Iruretagoyena & Orue, 2021), but is also negatively related to victimization (Ortega Barón & Carrascosa, 2018; Villalobos et al., 2016). In contrast, the absence of socioemotional support can intensify the consequences of victimization (Bartolomé Gutiérrez & Díaz Herráiz, 2020; El Keshky & Alganami, 2025; Valdés Cuervo, et al., 2018).

Along with these protective variables, life satisfaction also stands out, defined as a positive evaluation of one's own life (Martínez-Antón et al., 2007). Studies suggest that this satisfaction buffers the negative effects of victimization, partly thanks to perceived social support (Miranda et al., 2019; Rodríguez-Rivas et al., 2023; Zhang, et al., 2023). Specifically, community social support plays a crucial role in promoting life satisfaction, which fosters a sense of belonging that allows for the establishment of support networks that mitigate adverse experiences (Chen et al., 2017).

Likewise, socioemotional skills, such as resilience, are essential for protecting against victimization (Chirinos-Cazorla, and Araujo-Robles, 2022). Resilience, understood as the capacity to adapt to adversity, can be addressed from an individual perspective, associated with emotional strength and personal growth (Theron et al., 2013), or from a contextual perspective, as a product of interaction with the family, educational, and social environments (Rutter, 2007).

Several studies identify resilience as an effective protective resource in contexts of violence, acting as a shield against the psychological consequences of victimization in adolescents (Bitsika et al., 2022; Elfversson & Höglund, 2023). Furthermore, a negative relationship between resilience and victimization has been found, highlighting its preventive role (Márquez González et al., 2016; Rosado & Almeida, 2025). Likewise, resilience is positively related to life satisfaction (Betancourt-Salamanca et al., 2024; Cerezo et al., 2022; González-Aguilar, 2020), showing that resilient adolescents experience greater well-being due to their ability to adapt to difficult situations, including victimization.

This study explores how the development of socio-emotional competencies in adolescents provides them with protective resources against victimization, thereby fostering their emotional adjustment and overall well-being. The literature review provides evidence that school bullying is not an isolated phenomenon but a multidimensional problem with both individual and contextual roots. Although previous studies have identified its consequences and protective factors, they have not yet articulated an explanatory model that fully integrates the complexity of these interactions. To address this conceptual gap, our model is based on Bronfenbrenner's Ecological Systems Theory, which allows for a visualization of bullying dynamics not only at the individual level but also through their interaction with the systems in their environment.

From this perspective, school victimization is the result of interactions at multiple levels: the microsystem (direct ties with peers and family), the mesosystem (the connection between school and home), the exosystem (community policies and resources), the macrosystem (cultural norms), and the chronosystem (the time factor). Our model articulates these ideas, demonstrating how key variables fit into this framework: community social support reflects the influences of the meso- and exosystem, while resilience represents the individual's ability to adapt within the microsystem. In turn, life satisfaction and psychological distress synthesize the environment's impact on the student's well-being.

Given this theoretical background, the general objective of this study is to analyze school victimization in adolescents by examining individual and social variables. To achieve this, we established two specific objectives: 1) To analyze the existing relationships between school victimization, community social support, resilience, psychological distress, and life satisfaction; 2) To specify and contrast an explanatory model of school victimization that integrates these variables.

**Fig. 1:**
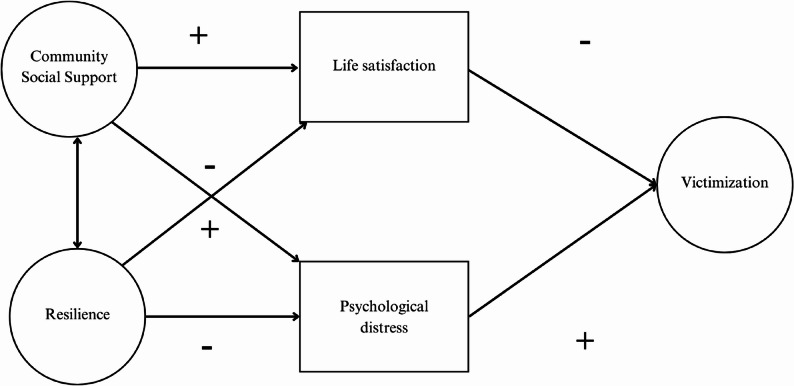
Hypothetical model of school victimization

The main contribution of this study is the construction of a model that, in addition to analyzing the relationship between victimization and psychosocial variables, allows us to understand the mechanisms that cause it. By integrating multidimensional factors, a more comprehensive and precise approach to a phenomenon as complex as victimization is offered. Based on these objectives, the following hypothetical model (Fig. 1) is proposed, comprised of the following factors:

- Factor 1: Community social support
- Factor 2: Resilience
- Factor 3: Life satisfaction
- Factor 4: Psychological distress
- Factor 5: Victimization

The hypothesis states that life satisfaction has a direct, negative relationship with school victimization, while psychological distress has a direct, positive relationship. Likewise, it is hypothesized that community social support has an indirect relationship with school victimization through life satisfaction and psychological distress. Similarly, it is hypothesized that resilience has an indirect relationship with school victimization through these same variables.

It is also relevant to consider gender differences in the experience of victimization, since gender roles and social expectations can influence how male and female adolescents perceive and confront school violence (Williams et al., 2017; Yang et al., 2022). This analysis is offered through a multi-group analysis.

## Method

### Participants

The participants were 1,687 adolescents (46% male, 54% female) aged 12 to 17 years (M = 13.65, SD = 1.14) from 13 secondary schools in the school region Costa Norte of Puerto Vallarta, Mexico. Participants were selected through a two-stage stratified cluster sampling (Santos et al., 2003). The sample is representative of a population of 14,759 students, with a sampling error of ± 2.5% and a confidence level of 95%, based on a sample size of 100 and a population variance of.50 (Morales, 2008).

### Instruments

#### Multidimensional peer-victimization scale (Mynard & Joseph, 2000)

It consists of 20 items organized into three factors: relational victimization, overt physical victimization, and overt verbal victimization. McDonald’s omega coefficient (ω) for the present study was 0.757 for the relational victimization subscale, 0.847 for the overt physical victimization subscale, 0.826 for the overt verbal victimization subscale, and 0.918 for the overall scale.

Exploratory factor analyses, conducted using the principal component analysis and Oblimin rotation, revealed a three-factor structure that explains 55.95% of the total variance. The first factor explains 43.49% of the variance; the second 6.55%; and the third 5.89%. Subsequently, a confirmatory analysis was performed, in which a three-factor model was evaluated for the 20 items using the maximum likelihood estimation method, due to the multivariate normality of the data (Mardia coefficient = 416.92, *p* >.05). The results indicated a good model fit [SBχ2 = 293.7139, df = 142, *p* <.001, CFI = 0.952, RMSEA = 0.031 (0.026, 0.036)].

#### Kessler psychological distress scale –K10– (Kessler & Mroczek, 1994)

The version used was the one adapted to Spanish by Alonso, Herdman, Pinto and Vilagut (2010), consisting of 10 items. It showed a coefficient (ω) of 0.889. The exploratory factor analysis, performed using the principal component analysis and Oblimin rotation, revealed a unifactorial structure that explains 50% of the total variance. The maximum likelihood estimation method was used due to the multivariate normality of the data (Mardia coefficient = 32.37, *p* >.05). The results indicated a good fit of the model to the data [SBχ2 = 120.9903, df = 30, *p* <.001, CFI = 0.983, RMSEA = 0.043 (0.035, 0.051)].

#### The satisfaction with life scale (Diener et al., 1985)

It consists of five items grouped into a single dimension. The McDonald’s omega coefficient (ω) obtained was 0.602. In the exploratory factor analysis, using the principal component analysis method and Oblimin rotation, a unidimensional structure was identified that explained 41.59% of the total variance, with the 5 items grouped into a single factor. A confirmatory analysis was performed, where a one-factor model was examined for the five items in the scale. Analyses using the maximum likelihood estimation method showed multivariate normality (Mardia coefficient = 5.19, *p* >.05). The model showed a good fit to the data [SBχ2 = 13.3204, df = 4, *p* <.05, CFI = 0.991, RMSEA = 0.037 (0.016, 0.060)].

#### Resilience scale RESI-M (Palomar Lever & Gómez Valdez, 2010)

It consists of 43 items distributed across five factors (fortitude and self-confidence, social competence, family support, social support, and structure). The McDonald’s omega coefficient (ω) for the present study was 0.942 for the fortitude and self-confidence subscale, 0.868 for the social competence subscale, 0.916 for the family support subscale, 0.889 for the social support subscale, 0.821 for the structure subscale, and 0.961 for the overall scale.

Exploratory factor analyses, performed using the principal component analysis method and with Oblimin rotation, revealed a five-factor structure that explains 57.52% of the total variance. The first factor explained 36.68% of the variance; the second factor explained 7.12%; the third factor 7.5%; the fourth 4%; and the fifth 3.48%. Subsequently, a confirmatory factor analysis was performed using the robust maximum likelihood estimation method for all analyses, due to the multivariate normality of the data (Mardia coefficient = 802.48, *p* >.05), showing a good model fit [S-B χ2 = 1397.9106, df = 840, *p* <.001, CFI = 0.950, RMSEA = 0.032 (0.029, 0.035)].

#### Perceived Community Support Questionnaire PCSQ (Gracia et al., 2002)

This scale consists of 24 items distributed across four factors, three of which were used in this study: community integration; community participation; and social support in informal systems. The McDonald’s omega coefficient (ω) for the present study was 0.607 for the community integration subscale, 0.660 for the community participation subscale, 0.756 for the social support in informal systems subscale, and 0.829 for the overall scale.

Exploratory factor analysis, performed using the principal component analysis method and Oblimin rotation, revealed a three-factor structure that explained 47% of the total variance. The first factor explained 29.42% of the variance; the second, 10.59%; and the third, 7.5%. Subsequently, a confirmatory analysis was performed. The robust maximum likelihood method was used for all analyses, since the data showed multivariate normality (Mardia coefficient = 171.21, *p* >.05). The results revealed a good fit of the model to the data [SBχ2 = 708.7472, df = 162, *p* <.001, CFI = 0.928, RMSEA = 0.043 (0.040, 0.047)].

### Procedure

Participants were selected through a two-stage stratified cluster sampling (Santos et al., 2003). From the list of educational centers in the region, 19 were selected, respecting the proportionality between public and private schools; in the second stage, classes were chosen from each school, taking into account proportionality by grade, shift, and school size. Schools and tutors were informed, ensuring informed consent, anonymity, and confidentiality. During administration, adolescents were informed that their participation was voluntary and that they could withdraw from the process of completing the questionnaire at any time; 16 students chose not to participate in the study, and the response rate was 99%.

The study adhered to the ethical principles established in the Declaration of Helsinki, ensuring data protection and the right to withdraw at any time. The study was approved by the Research Ethics Committee of the Centro Universitario de la Costa (CEICUC, for its abbreviation in Spanish) on April 28, 2017.

### Data analysis

An explanatory, cross-sectional and ex post facto design was used. Data coding and analysis were performed using SPSS version 22. An explanatory, cross-sectional, and ex post facto design was used. Data coding and analysis were carried out using SPSS version 22. The regression imputation method was applied to handle missing values, provided that they did not exceed 20% of a scale (Norman et al., 1998; Useche et al., 2006); participants were excluded if they presented more than two scales with over 20% missing data.

Univariate outliers were detected using standardized scores, with values ​​greater than 4 considered outliers (Hair et al., 2018). Multivariate values ​​were identified using the Mahalanobis distance (Tabachnick et al., 2019). Eleven participants were excluded from the sample, seven due to missing values and four due to outliers. An exploratory Pearson correlation analysis was performed to determine the relationship between the study variables. For structural equation modeling (SEM), EQS 6.1 and R 4.5.1 running in RStudio 2025.05.1 were employed.

## Results

### Preparation of the general model

To analyze the relationship between the study variables and school victimization, Pearson correlations were performed, which showed statistically significant relationships between all the variables analyzed, as shown in Table 1. Relational victimization correlates positively with physical victimization (*r* =.709, *p* <.01), verbal victimization (*r* =.838, *p* <.01) and psychological distress (*r* =.377, *p* <.01); and is negatively and significantly correlated with community integration (*r* = -.151, *p* <.01), community participation (*r* = -.071, *p* <.01), social support in informal systems (*r* = -.125, *p* <.01), fortitude and self-confidence (*r* = -.138, *p* <.01), social competence (*r* = -.104, *p* <.01), family support (*r* = -.188, *p* <.01), social support (*r* = -.137, *p* <.01), structure (*r* = -.118, *p* <.01) and life satisfaction (*r* =.−194, *p* <.01).

**Table 1:** Pearson correlations

	1	2	3	4	5	6	7	8	9	10	11	12	13
1. RV	1												
2. OPV	.709^**^	1											
3. OVV	.838^**^	.720^**^	1										
4. CI	-.151^**^	-.113^**^	-.147^**^	1									
5. CP	-.071^**^	-.032^**^	-.098^**^	.443^**^	1								
6. SSIS	-.125^**^	-.075^**^	-.144^**^	.557^**^	.557^**^	1							
7. FSC	-.138^**^	-.122^**^	-.132^**^	.250^**^	.247^**^	.290^**^	1						
8. SC	-.104^**^	-.060^*^	-.086^**^	.234^**^	.233^**^	.288^**^	.641^**^	1					
9. FS	-.188^**^	-.157^**^	-.203^**^	.282^**^	.259^**^	.301^**^	.531^**^	.357**	1				
10. SS	-.137^**^	-.141^**^	-.125^**^	.242^**^	.230^**^	.297^**^	.565^**^	.477**	.627^**^	1			
11. ST	-.118^**^	-.067^**^	-.128^**^	.244^**^	.274^**^	.316^**^	.605^**^	.511**	.549^**^	.592^**^	1		
12. LS	-.194^**^	-.189^**^	-.193^**^	.262^**^	.161^**^	.222^**^	.397^**^	.293**	.438^**^	.343^**^	.319^**^	1	
13. PD	-.377^**^	.244^**^	.365^**^	-.207^**^	-.231^**^	-.232^**^	-.301^**^	-.168**	-.421^**^	-.229^**^	-.264^**^	-.345^**^	1

*RV* Relational Victimization, *OPV* Overt Physical Victimization, *OVV* Overt Verbal Victimization, *CI* Community Integration, *CP* Community Participation, *SSIS* Social Support in Informal Systems, *FSC* Fortitude and Self-Confidence, *SC* Social Competence, *FS* Family Support, *SS* Social Support, *ST* Structure, *LS* Life Satisfaction, *PD* Psychological Distress

^**^The correlation is significant at the 0.01 level (two-tailed)

^*^The correlation is significant at the 0.05 level (two-tailed)

Regarding the second objective, aimed at analyzing the direct and indirect influence of individual and social factors on school victimization, the estimated model was tested using the structural equation technique (Bentler & Wu, 2002). The structural model evaluated consisted of five factors, two of which are observed variables (composed of a single indicator) and three latent factors. The latent factors present in the model are social support with three indicators: community integration, community participation, and social support in informal systems; resilience with five indicators: fortitude and self-confidence, social competence, family support, social support and structure; and school victimization with three dimensions: relational victimization, overt physical victimization, and overt verbal victimization. The observed variables are psychological distress and life satisfaction. The indicators or dimensions: community integration, fortitude and self-confidence, and relational victimization were set at 1 by the program during the estimation.

To assess the model's fit to the data, the following scheme was proposed: community social support exerts an indirect effect on victimization, mediated in parallel by life satisfaction and psychological distress; resilience exerts an indirect effect on victimization, mediated in parallel by psychological distress and life satisfaction. In addition, a correlation between the community social support and resilience factors was specified.

To determine the goodness of fit of the model and the statistical significance of the coefficients, the DWLS (Diagonally Weighted Least Squares) estimator was employed, since the normalized Mardia coefficient indicated deviation from normality in the data (Normalized Mardia Coefficient = 61.38, *p* <.05). This choice is appropriate given the nature of the variables and the sample (Forero et al., 2009). Due to the sensitivity of the chi-square coefficient to sample size, alternative fit indices were used (Hair et al., 2018). The indices obtained in the calculation of the model indicate a good fit to the data: [S-B χ2 = 151.07, gl = 60, *p* <.001, CFI = 0.989, TLI = 0.986, RMSEA = 0.031 (0.025, 0.037)]. The unstandardized and standardized solutions are presented in Table 2. Acceptable values ​​for the CFI and TLI, are.95 or higher, while values ​​for the RMSEA are.05 or lower (Hu & Bentler, 1999), indicating a good fit to the data. This model accounts for 16.2% of the variance in victimization.

**Table 2:** Non-standardized and standardized solutions

Unstandardized solution 1	Community Social Support (F1)	Resilience (F2)	Life Satisfaction	Psychological Distress	Victimization (F3)
Community Integration	1^a^				
Community Participation	.920 (.042)				
Social Support in Informal Systems	1.057 (.047)				
Fortitude and Self-Confidence		1^a^			
Social Competence		.875 (.035)			
Family Support		1.252 (.047)			
Social Support		.963 (.037)			
Structure		1.034 (.039)			
Life Satisfaction	.112 (.042)	.544 (.049)			
Psychological Distress	−0.587 (.052)	-.587 (.052)			
Relational Victimization					1^a^
Overt Physical Victimization					.642 (.040)
Overt Verbal Victimization					1.019 (.063)
			-.161	.301	
Victimization (F3)			(.023)	(.022)	
	Community Social Support (F1)	Resilience (F2)	Life Satisfaction	Psychological Distress	Victimization (F3)
Community Integration	.700				
Community Participation	.658^***^				
Social Support in Informal Systems	.800^***^				
Fortitude and Self-Confidence		.783			
Social Competence		.633^***^			
Family Support		.772^***^			
Social Support		.733^***^			
Structure		.743^***^			
Life Satisfaction	.085**	.407^***^			
Psychological Distress	-.175***	-.316^***^			
Relational Victimization					.931
Overt Physical Victimization					.737^***^
Overt Verbal Victimization					.937^***^
Victimization (F3)			-.134^***^	.351^***^	

Standardized solution	Community Social Support (F1)	Resilience (F2)	Life Satisfaction	Psychological Distress	Victimization (F3)
Community Integration	.700				
Community Participation	.658^***^				
Social Support in Informal Systems	.800^***^				
Fortitude and Self-Confidence		.783			
Social Competence		.633^***^			
Family Support		.772^***^			
Social Support		.733^***^			
Structure		.743^***^			
Life Satisfaction	.085**	.407^***^			
Psychological Distress	-.175***	-.316^***^			
Relational Victimization					.931
Overt Physical Victimization					.737^***^
Overt Verbal Victimization					.937^***^
Victimization (F3)			-.134^***^	.351^***^	

Factor loadings greater than 0.45 (reference point)

Z > + 1.96; *p* <.05 *; Z > + 2.56, *p* <.01 **, Z > + 3.29, *p* <.001 ***

^1^Robust standard errors. All coefficients are significant (*p* <.001)

^a^Set to 1 during estimation

**Fig. 2:**
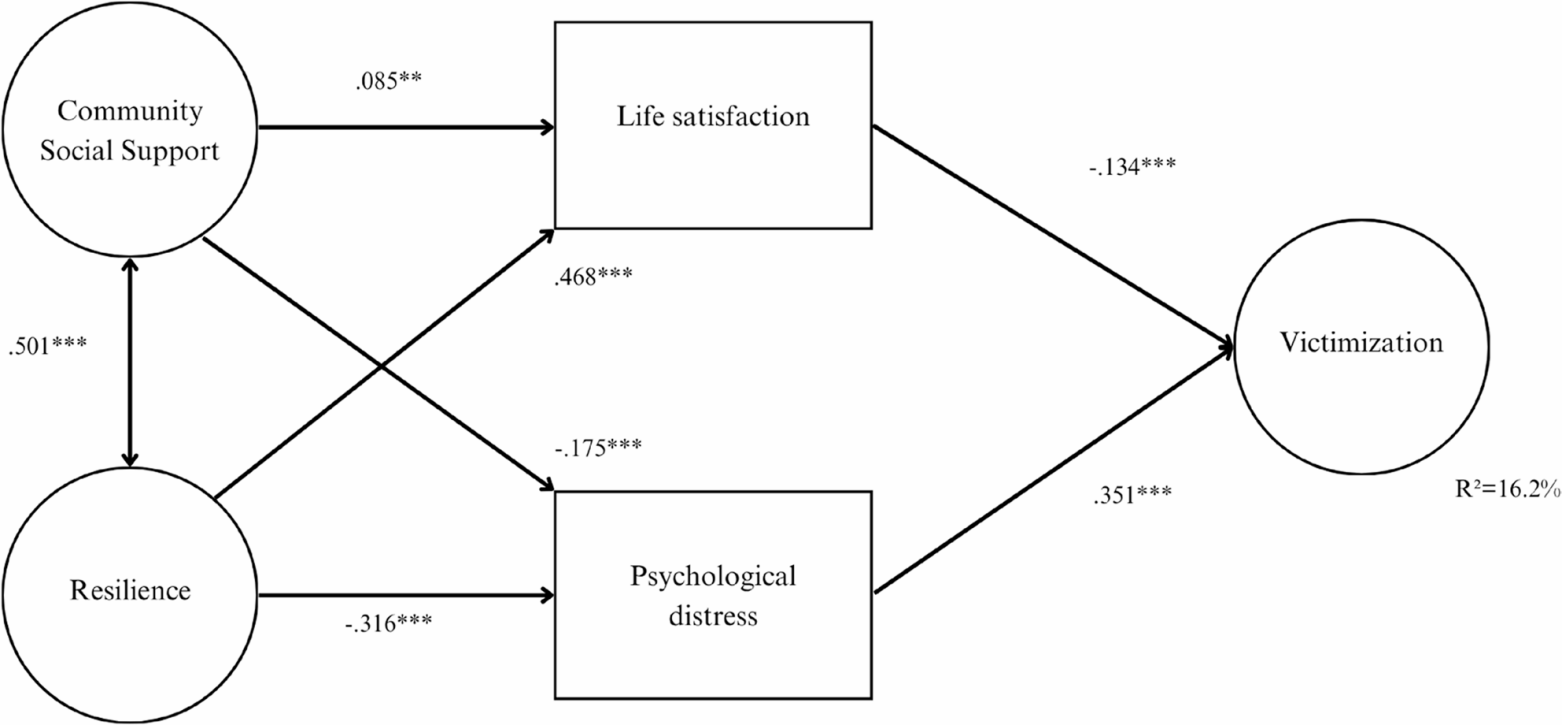
Explanatory model of school victimization

Figure 2 shows the graphical representation of the calculated structural model, the corresponding standardized coefficients and their associated probability.

The calculated model shows direct relationships in the prediction of school victimization. The results show a significant positive relationship between psychological distress and school victimization (βstd =.351, *p* <.001). Life satisfaction is also negatively related to school victimization (βstd = -.134, *p* <.001).

Furthermore, the model shows statistically significant indirect effects in predicting school victimization. Community social support and resilience are correlated (βstd =.501; *p* <.001). Community social support influences life satisfaction directly and positively (βstd =.085; *p* <.01) and, at the same time, influences psychological distress directly and negatively (βstd = -.175; *p* <.001).

Resilience influences life satisfaction in a direct and positive way (βstd =.468; *p* <.001) and also influences psychological distress in a direct and negative way (βstd = -.316; *p* <.001).

### Multigroup analysis

Once the model was calculated with the general sample, a multigroup analysis was performed to test the model's goodness-of-fit and structural invariance across gender by comparing two models. Parameters were progressively constrained following a sequence of measurement invariance: configural, metric, and scalar, up to structural invariance, fixing regressions and covariances. Models were compared using the absolute change criterion (Δ ≤ 0.01) in fit indices (CFI, TLI, RMSEA, SRMR) (Putnick & Bornstein, 2016), estimated at each level of constraint with the DWLS estimator (Table 3).

**Table 3:** Multigroup analysis, structural invariance

	CFI	TLI	RMSEA	SRMR	Δ CFI	Δ TLI	Δ RMSEA	Δ SRMR
Configural	.989	.986	.031	.043				
Metric	.980	.976	.040	.052	.009	.010	.009	.008
Scalar	.969	.967	.047	.057	.010	.009	.007	.005
Structural (Regression)	.969	.967	.047	.057	.000	.000	.000	.000
Covariances	.965	.963	.050	.063	.005	.004	.003	.006

Subsequently, the model was replicated in subsamples of men and women to compare the explained variance in victimization in each group, yielding R^2^ = 22.5% for men and R^2^ = 17.2% for women.

## Discussion

The results of this study offer a comprehensive understanding of the psychosocial factors that influence school victimization, integrating individual factors such as psychological distress, life satisfaction, and resilience, along with social factors such as community support. The articulation of these variables allowed for the construction of an explanatory model that not only considers the direct relationships between them but also sheds light on the indirect mechanisms through which they interact to influence the experience of victimization.

The findings show that school victimization correlates positively with psychological distress, consistent with previous research (Balluerka et al., 2022; Gini et al., 2024; Vega-Cauich & Euan-Catzin, 2020). In contrast, life satisfaction shows a negative relationship with school victimization, in line with previous studies (Miranda et al., 2019; Villalobos et al., 2016), confirming the protective role of life satisfaction against school victimization.

The present study offers evidence that both resilience and community social support directly influence life satisfaction and, at the same time, contribute to the reduction of psychological distress, which is consistent with previous studies (Ayllón-Salas & Fernández-Martín, 2024; Balluerka et al., 2022; Bartolomé Gutiérrez & Díaz Herráiz, 2020; Kwon et al., 2024).

An important contribution of the model is the indirect effect of community social support and resilience on victimization, mediated by life satisfaction and psychological distress. This finding emphasizes the importance of social and personal resources in preventing victimization experiences (Bitsika et al., 2022; Elfversson & Höglund, 2023; Zhang et al., 2023) and confirms that community social support is a crucial factor in building environments that promote well-being and reduce vulnerability to school victimization.

The structural model presented constitutes a significant contribution, integrating multiple psychosocial variables that allow for further understanding of how individual and social factors interact in the experience of victimization. Furthermore, the multigroup analysis revealed differences in victimization dynamics by gender.

Gender differences in adolescent school victimization are consistent: males tend to experience higher levels of physical and direct victimization, while females more frequently face relational forms (Hosozawa et al., 2021; Yang et al., 2022; Zhou et al., 2024). In this study, the model was invariant, and therefore applicable, for both men and women. However, it showed substantially greater explanatory power for victimization among males, suggesting that, in the case of females, other, more influential variables not included here may be at play. These findings emphasize the need to design gender-sensitive interventions that respond to the particularities of each group.

### Limitations and future lines of research

Among the limitations of the study is its cross-sectional nature, which does not allow testing the stability of the model across different time periods or changing contexts. Therefore, future longitudinal research is recommended to examine how these variables evolve over time. Furthermore, it would be pertinent to explore the replicability of these findings in different cultural contexts. Likewise, the use of self-report instruments may introduce response biases due to social desirability; however, it has been confirmed that self-reports have acceptable reliability and validity in the measurement of risk behaviors (Buelga et al., 2015). Additionally, the study did not incorporate the complex sampling design into the statistical modeling, which may have underestimated standard errors; future research should include sampling corrections to improve the robustness and validity of the results.

### Practical implications

In practical terms, the results highlight the importance of implementing intervention programs that strengthen both formal and informal support networks, promote resilience skills, and improve adolescents' perceived subjective well-being. These interventions should include strategies aimed at developing socioemotional skills, fostering personal confidence, and building cohesive school environments that enhance collective well-being.

## Conclusions

In conclusion, the study of school victimization from an integrative perspective of psychosocial variables offers valuable guidelines for the design of multidimensional interventions, aimed not only at mitigating the immediate effects of victimization, but also at strengthening the personal and social resources of the teenage students, contributing to their well-being and development. Finally, this approach reaffirms that both the prevention and response to school victimization must consider students and teachers as a collective, since interventions focused solely on victims or aggressors are insufficient in the face of the complexity of this phenomenon.

## Data Availability

The datasets generated and/or analysed during the current study are not publicly available due to ethical restrictions and the protection of participants’ privacy, but are available from the corresponding author on reasonable request.

## Abbreviations

CFI: Comparative Fit Index
CI: Community Integration
CP: Community Participation
DWLS: Diagonally Weighted Least Squares
EQS: Statistical software used to analyze SEM models
FS: Family Support
FSC: Fortitude and Self-Confidence
IFI: Incremental Fit Index
K10: Kessler Psychological Distress Scale
LM: Lagrange Multipliers
LS: Life Satisfaction
M: Mean
N: Total number
NNFI: Non-Normed Fit Index
OPV: Overt Physical Victimization
OVV: Overt Verbal Victimization
PD: Psychological Distress
r: Pearson correlation coefficient
REDIM: Network for Children's Rights in Mexico
RESI-M: Resilience Scale
RMSEA: Root Mean Square Error of Approximation
RV: Relational Victimization
S-B χ2: Satorra-Bentler Chi-square
SC: Social Competence
SD: Standard deviation
SEM: Structural Equation Modeling
SRSR: Standardized Root Mean Square Residual
SS: Social Support
SSIS: Social Support in Informal Systems
ST: Structure
TLI: Tucker–Lewis Index
UNICEF: United Nations Children's Fund
Z: Standard value that indicates how many standard deviations a value is above or below the mean
Βstd: Coeficiente beta estandarizado
ω: McDonald’s omega coefficient
